# Interplay Between Membrane Permeability and Enzymatic Barrier Leads to Antibiotic-Dependent Resistance in *Klebsiella Pneumoniae*

**DOI:** 10.3389/fmicb.2018.01422

**Published:** 2018-06-29

**Authors:** Marie-Helene Nicolas-Chanoine, Noémie Mayer, Kathleen Guyot, Estelle Dumont, Jean-Marie Pagès

**Affiliations:** ^1^Service de Microbiologie, Hôpital Beaujon, AP-HP, Clichy, France; ^2^Faculté de Médecine D. Diderot, Paris, France; ^3^Institut National de la Santé et de la Recherche Médicale UMR 1137, Université Paris 7, Paris, France; ^4^UMR_MD1, Aix-Marseille Univ, IRBA, Marseille, France

**Keywords:** *Klebsiella pneumoniae*, efflux pump, porins, beta-lactamases, resistance mechanism interplay, ceftolozane+tazobactam, ceftazidime+avibactam

## Abstract

The interplay between membrane permeability alterations and the enzymatic barrier contributes to *Klebsiella pneumoniae* multidrug resistance. We assessed the specific effect of the efflux levels of the main efflux pumps (AcrAB and OqxAB), alone and associated with the loss of the main porins (OmpK35 and OMPK36), on the activity of various antibiotics by constructing a set of *K. pneumoniae* isogenic strains, including strains with plasmid-mediated β-lactamases (DHA-1, CTX-M-15, and OXA-48). The two pumps contributed to intrinsic chloramphenicol resistance and AcrAB to that of nalidixic acid and cefoxitin, whereas they had no impact on the activity of the other 11 antibiotics tested. We confirmed the expulsion of these three antibiotics by the two overproduced pumps and that of tigecycline by overproduced AcrAB, and showed that overproduced AcrAB also expelled ertapenem, piperacillin, ceftolozane, and ceftazidime. The sole loss of porins did not significantly affect the activity of the tested antibiotics, except ertapenem. The effect of efflux increases and porin loss on β-lactam activity was the highest in plasmid-mediated β-lactamase-producing strains. Thus, DHA-1-producing strains became non-susceptible (NS) to (i) ertapenem when there was an increase in efflux or porin loss, (ii) imipenem and ceftazidime+avibactam when the two mechanisms were associated, and (iii) temocillin when AcrAB was overproduced. The CTX-M-15-producing strains became NS to (i) ertapenem when there was no porin, (ii) ceftolozane+tazobactam when there was either overproduced OqxAB or porin loss, and (iii) temocillin when AcrAB was overproduced. OXA-48-producing strains known to be NS to temocillin were also NS to ceftolozane and they became NS to imipenem when the two pumps were overproduced or there was porin loss. Overall, this study shows that the balance between influx and efflux differentially modulates the activity of the tested antibiotics, an important point for evaluating the activity of future antibiotics or new combinations.

## Introduction

*Klebsiella pneumoniae* is a pathogen responsible for a wide range of nosocomial infections (Podschun and Ullmann, 1998; Wyres and Holt, 2016). Moreover, it is the enterobacterial species in which plasmid-mediated resistance to extended-spectrum β-lactams related to extended-spectrum β-lactamases (ESBL) (Jarlier et al., 1988), cephalosporinases (Park et al., 2013; Freitas et al., 2014), and carbapenemases (Nordmann et al., 2011; Robert et al., 2014) first emerged and then became widely disseminated. Thus, it has been included in the ESKAPE group, which clusters the main bacterial species *(*i.e., *Enterococcus faecium, Staphylococcus aureus, K. pneumoniae, Acinetobacter baumannii, Pseudomonas aeruginosa*, and *Enterobacter* species) with a propensity for multidrug resistance (Pendleton et al., 2013). It is also the enterobacterial species for which a noticeable reduction of antibiotic accumulation, related to overexpression of efflux pumps and porin alteration, has been commonly reported, notably in carbapenem-resistant isolates devoid of carbapenemase (Hasdemir et al., 2004; Dahmen et al., 2012; López-Camacho et al., 2014). Thus, multidrug-resistant clinical isolates of *K. pneumoniae* constitute threatening nosocomial pathogens as the number of antibiotics active against them become increasingly limited.

The aim of this study was to decipher the contribution of each mechanism, alone and together, to the resistance to both old and recently marketed antibiotics of various families. We thus constructed ~40 new variants from *K. pneumoniae* isogenic strains that have already been molecularly and phenotypically characterized concerning the production of the chromosomally encoded OqxAB and AcrAB pumps and synthesis of the general porins OmpK35 and OmpK36 (Bialek et al., 2010; Bialek-Davenet et al., 2013, 2015). These new variants were specifically designed to evaluate the effect of various levels of production of each efflux pump, alone and associated with each other, as well as that of various porin alterations, alone and associated with the overproduction of various efflux pumps. They were also designed to determine how these outer membrane permeability changes modulate the level of β-lactam resistance associated with the production of various β-lactamases. Moreover, several new variants were specifically designed to assess the previously suggested role of the regulators of efflux pump gene expression on the production of general porins in *K. pneumoniae* (De Majumdar et al., 2013, 2015): RamA and RamR, which activate or repress *acrAB* gene expression, respectively, and RarA and OqxR, which activate or repress, *oqxAB* gene expression, respectively (Bialek-Davenet et al., 2013, 2015).

Our protocol showed that membrane permeability alterations and the enzymatic barrier leads to antibiotic-dependent resistance in *K. pneumoniae*, an important point for evaluating the activity of future antibiotics or new combinations.

## Materials and methods

### Bacterial strains and plasmids

Previously published isogenic *K. pneumoniae* strains (Table S1) were used in this study to construct and characterize additional variants. *K. pneumonia*e ATCC13883 was used as a control strain. Clinical isolate-extracted plasmids encoding various β-lactamases [CTX-M-15 (combined with OXA-1 and TEM-1), DHA-1, and OXA-48] and the constructed plasmids presented in Table S1 were used to transform some variants.

### *In Vitro* selection of antibiotic-resistant mutants

Mutants were selected using a previously described procedure (Bialek et al., 2010; Bialek-Davenet et al., 2011). Briefly, 5 μL of an overnight culture were transferred into 9 mL fresh Mueller Hinton (MH) II broth containing the antibiotic selector at 0.25x the minimum inhibitory concentration (MIC) for the strain tested and incubated for 18 h at 37°C. Such growth conditions were repeated until mutants were obtained from overnight culture aliquots plated between each broth culture cycle on MH agar containing 4x the original MIC. Ertapenem (Merck Sharpe and Dohme, Clermont-Ferrand, France) was used to obtain mutants with porin alterations, whereas tigecycline (Pfizer, Paris, France), previously shown to be a substrate of solely the efflux pump AcrAB was used to select mutants which overexpressed the *acrAB* genes associated with mutations in the *ramR* gene encoding the repressor of *acrAB* expression (Bialek-Davenet et al., 2013).

### Analysis of gene sequences

The presence of mutations in the ompK35, ompK36, and ramR genes was assessed by PCR and sequencing, as previously described (Bialek-Davenet et al., 2011). The putative IS identified in the ompK36 gene promoter was characterized using ISFinder (http://www-is.biotoul.fr).

### Gene replacement experiments

Inactivation of the chromosomal *acrB* gene was achieved following a strategy adapted from Datsenko and Wanner, as previously described (Bialek-Davenet et al., 2015), whereas inactivation of the *rarA* gene was carried out by SMALTIS (www.smaltis.fr).

### Complementation with the wild type *oqxR* and *ramR* genes

Complementation with the wild type *oqxR* and *ramR* genes was performed using (i) the already available recombinant plasmids, pSC-A-amp/kan-*oqxR*-ATCC and pSC-A-amp/kan-*ramR*-ATCC (Table S1), when the complemented strains were susceptible to kanamycin, and (ii) the recombinant plasmid pBBRMCS-III-*oqxR*-ATCC (Table S1), constructed in this study, when the complemented strains were resistant to kanamycin (strains with an *acrB* gene knockout). Briefly, the 677-bp wild type *oqxR* gene of strain ATCC 13883 was amplified using primers XbaI-oqxRF and XhoI-oqxRR (Table S1), digested with *Xb*aI and *Xho*I, and ligated to pBBRMCS1-III digested with the same restriction enzymes. After verification of the construct by sequencing, the recombinant plasmid pBBRMCS-III-*oqxR*-ATCC was electroporated into competent strains and the transformants were selected on Luria Bertani (LB) agar plates containing 10 mg/L tetracycline (Sigma-Aldrich, Saint Quentin Fallavier, France).

### Analysis of gene expression by real-time RT-PCR

Real-time reverse transcription (RT)-PCR for analyzing the expression of the *ompK35, ompK36, acrB, oqxB, ramA*, and *rarA* genes was carried out and interpreted as previously described (Bialek-Davenet et al., 2015). Briefly, after RNA extraction and DNaseI pre-treatment, total RNA concentrations were determined using a NanoDrop Spectrophotometer and adjusted to 100 mg/L. Amplifications were performed in duplicate on a LightCycler using the one-step LightCycler® RNA Master SYBR Green I kit (Roche Applied Science, Meylan, France). Data were analyzed using the 2^−ΔΔCt^ method, in which the *rpoB* gene was chosen as a reference, and strain ATCC13883 as a calibrator. All primers used are listed in Table S1.

### Immunocharacterization of outer membrane proteins

Outer membrane proteins were prepared by ultrasonic treatment as previously described (Philippe et al., 2015). Each strain was grown in three different broth media: MH II, low osmotic-strength nutrient broth (NB), to increase porin *ompK35* gene expression, and high osmotic-strength nutrient broth containing sorbitol (NBS), to increase *ompK36* gene expression. Protein extracts of the final preparations were electrophoresed on sodium dodecyl sulfate polyacrylamide gels (acrylamide 10% W/V; SDS 0.1% W/V) and electro-transferred onto nitrocellulose membranes, which were incubated, as previously described (Philippe et al., 2015), in the presence of polyclonal antibodies directed against denatured OmpA and denatured OmpC and OmpF porins, known as OmpK36 and OmpK35, in *K. pneumoniae*, respectively. The detection of antigen-antibody complexes was performed with goat anti-rabbit horseradish-peroxidase conjugated immunoglobulin G secondary antibodies and a chemiluminescent kit (BioRad, Marne La Coquette, France).

### Antibiotic susceptibility testing

Antibiotic susceptibility was determined in triplicate by the broth MH II dilution method, according to the guidelines of the European Committee on Antimicrobial Susceptibility Testing (EUCAST and ESCMID, 2003) and interpreted according to the 2016 EUCAST /French Antibiogram Committee of the French Microbiological Society (CASFM) recommendations (http://www.sfm-microbiologie.org). Differences between MICs were considered to be significant for two or more dilutions (i.e., a 4-fold MIC variation).

Nalidixic acid (Sigma), chloramphenicol (Sigma), cefoxitin (Merck Sharpe and Dohme), and ertapenem (Merck Sharpe and Dohme), for which the activity was previously shown to be reduced in association with efflux pump expression and/or porin alteration (Bialek et al., 2010; Bialek-Davenet et al., 2015) were first tested to phenotypically characterize the constructed variants. The following antibiotics were then tested on all characterized variants, including variants producing CTX-M-15, associated with OXA-1 and TEM-1 and DHA-1 and OXA-48: imipenem (Merck Sharpe and Dohme), temocillin (Eumedica s.a, Manage, Belgium), piperacillin alone and associated with 4mg/L tazobactam (Sigma), ceftolozane alone and associated with 4mg/L tazobactam (Merck Sharp & Dohme Corp., North Wales, Pennsylvania, USA), ceftazidime alone and associated with 4 mg/L avibactam (AstraZeneca, Cambridge, UK), tigecycline (Pfizer), and colistin (Sigma).

## Results

### Effect of various levels of the OqxAB and AcrAB efflux pumps on nalidixic acid, chloramphenicol, and cefoxitin activity

We confirmed (Table 1) the previously published cross-resistance to nalidixic acid, chloramphenicol, and cefoxitin observed in strain KPBj1 E+, which overproduces the OqxAB pump, due to a mutation in the negative regulator OqxR, and normally produces the AcrAB pump (Table S1). This phenotype disappeared when strain KPBj1 E+ T_*oqxR*−ATCC_ expressed normal levels of OqxAB following complementation with the *oqxR* gene of strain ATCC13883, (Table 1). The MICs of nalidixic acid and chloramphenicol were not significantly different from those observed in strain KBBj E+ when the *acrB* gene was deleted (strain KPBj1 E+ Δ*acrB*), whereas the MIC of cefoxitin was significantly lower (Table 1). This suggests that cefoxitin is expelled from the bacteria when the AcrAB pump is normally produced. However, the MIC of nalidixic acid in strain KPBj1 E+ Δ*acrB* T_*oqxR*−ATCC_ was significantly lower than that in strain KPBj1 E+ T_*oqxR*−ATCC_ (Table 1). This result suggests that nalidixic acid is also expelled by the normally produced AcrAB pump.

**Table 1:** Expression of OqxAB and AcrAB efflux pumps and effect on antibiotic susceptibility of isogenic strains of *Klebsiella pneumoniae*.

**Strain**	Dynamic expression (fold change)^*^		**MIC (mg/L)**		
	***oqxB***	***acrB***	NAL (≤ 16, > 16)^**^	CMP (≤ 8, > 8)^**^	FOX (≤ 8, > 16)^**^
ATCC13883	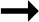 (1)	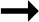 (1)	4	4	4
KPBj1 E+	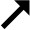 (85 ± 6.14)	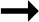 (0.96 ± 0.29)	64	128	16
KPBj1 E+ T_*oqxR*−ATCC_	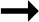 (4.04 ± 0.14)	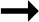 (1.21 ± 0.1)	4	4	4
KPBj1 E+ Δ*acrB*	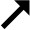 (74.9 ± 26.6)	0	128	128	4
KPBj1 E+ Δ*acrB* T_*oqxR*−ATCC_	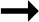 (4.89 ± 1.03)	0	1	2	2
KPBj1 E+ *ramR*A22V	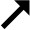 (390 ± 112)	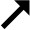 (3.41 ± 0.86)	256	128	128
KPBj1 E+ *ramR*A22V T_*oqxR*−ATCC_	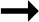 (2.02 ± 0.58)	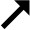 (6.50 ± 2.42)	64	128	64
KPBj1 E+ Δ*rarA*	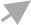 (33.1 ± 11.5)	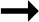 (0.95 ± 0.03)	16	32	8
KPBj1 E+ Δ*rarA ramR*59stop	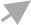 (65.03 ± 8.97)	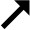 (2.85 ± 0.32)	128	256	32
KPBj1 Rev	0	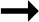 (1.15 ± 0.15)	4	4	4
KPBj1 Rev Δ*acrB*	0	0	1	1	2
KPBj1 M3 Lev	0	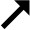 (3.74 ± 0.37)	32	32	64
KPBj1 M3 Lev T_*ramR*−ATCC_	0	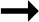 (1.34 ± 0.14)	4	4	4
KPBj1 M3 Lev Δ*acrB*	0	0	2	1	1
KPBj1 M3 Lev Δ*ramA*	0	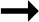 (1.075 ± 0.195)	4	2	4

* qRTPCR with strain ATCC13883 as calibrator; NAL, nalidixic acid; CMP, chloramphenicol; FOX, cefoxitin;

** *: breakpoints. Arrows indicate the level of gene expression: 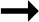, normal; 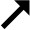: high, 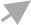: intermediate; 0, undetectable*.

The MICs of both nalidixic acid and cefoxitin increased significantly, but not that of chloramphenicol, when overproduction of the OqxAB pump was associated with overproduction of the AcrAB pump, due to a mutation in the negative regulator RamR (strain KPBj1 E+ *ramR*A22V) (Table 1). This result may reflect an additive effect of the two pumps to expel nalidixic acid and cefoxitin. Overproduction of the AcrAB pump and normal production of the OqxAB pump (strain KPBj1 E+ *ramR*A22V T_*oqxR*−ATCC_) resulted in nalidixic acid and chloramphenicol MIC values identical to those obtained when only OqxAB was overproduced (strain KPBj1 E+), whereas that of cefoxitin was significantly higher (64 mg/L vs. 16 mg/L) (Table 1).

Previous studies have shown that OqxR negatively regulates not only the expression of the *oqxAB* genes but also that of the *rarA* gene encoding RarA, an activator of *oqxAB* gene expression (Veleba et al., 2013; Bialek-Davenet et al., 2015). Deletion of the *rarA* gene resulted in an intermediate level of *oqxB* transcription by strain KPBj1 E+ Δ*rarA* relative to that of strain KPBj1 E+ overexpressing both the *oqxB* and *rarA* genes (Table 1). This suggests that RarA boosts the expression of the *oqxAB* genes, which is already elevated when OqxR no longer represses *oqxAB* gene expression. The intermediate level of *oqxAB* expression in strain KPBj1 E+ Δ*rarA* resulted in intermediate MIC values of the three antibiotics relative to those of strains KPBj1 E+ and KPBj1 E+ T_*oqxR*−ATCC_ (Table 1). This shows the presence of a relationship between the level of *oqxAB* expression and the MIC values of the three antibiotics. The nalidixic acid, chloramphenicol, and cefoxitin MICs were significantly higher in strain KPBj1 E+ Δ*rarA ramR*59stop, which produced the OqxAB pump at an intermediate level and overproduced the AcrAB pump due to a mutation in the negative regulator RamR (Table 1), than strain KPBj1 E+ Δ*rarA*.

Deletion of the *acrB* gene from strain KPBj1 Rev, devoid of the *rarA* gene and the *oqxRAB* operon, and normally producing the AcrAB pump (Table S1), resulted in nalidixic acid and cefoxitin susceptibility that was identical to that observed in strain KPBj1 E+ Δ*acrB* T_*oqxR*−ATCC_ (normal production of OqxAB and no production of AcrAB) (Table 1). This confirms that nalidixic acid and cefoxitin are expelled by the normally produced AcrAB pump, but not by the normally produced OqxAB pump. The MIC of chloramphenicol in strain KPBj1 Rev Δ*acrB* was lower (1 mg/L) than that obtained in strain KPBj1 Rev (4 mg/L) (Table 1), suggesting that chloramphenicol is also expelled by the normally produced AcrAB pump. However, the significantly lower MIC observed in strain KBj1 M3 Lev (32 mg/L) than strain KPBj1 E+ *ramR*A22V T_*oqxR*−ATCC_ (128 mg/L) suggests that chloramphenicol is also expelled by the normally produced OqxAB pump. Indeed, these two strains, which overproduce the AcrAB pump, differ in their production of OqxAB: no production by strain KBj1 M3 Lev and normal production by strain KPBj1 E+ *ramR*A22V T_*oqxR*−ATCC_ (Table 1). The higher cefoxitin MIC observed in strain KPBj1 M3 Lev (64 mg/L) than strain KPBj1 E+ (16 mg/L) (Table 1) suggests a higher affinity of cefoxitin to AcrAB than OqxAB.

Deletion of the *ramA* gene from strain KPBj1 M3 Lev, in which *acrB* and *ramA* are overexpressed, resulted in the both decrease in the *acrB* transcription and the MICs of nalidixic acid, chloramphenicol, and cefoxitin to reach the values obtained when the *acrB* gene is normally produced (Table 1). This finding clearly shows that overproduction of the AcrAB pump is controlled by RamA production.

### Molecular and phenotypic characterization of the OmpK35 and OmpK36 porins in mutants selected under ertapenem pressure

The sequences of the *ompK35* and *ompK36* genes were determined from parental strains (sequences called A and B for *ompK35* and *ompK36*, respectively) and seven mutants selected under ertapenem pressure (Table 2). There was no mutation in the *ompK35* gene of any mutant, but one (strain KPBj1 Rev P-), whereas there were various genetic events in the *ompK36* gene of all, but one, (strain KPBj1 Rev P-) (Table 2). The single mutated *ompK35* gene and four mutated *ompK36* genes had premature stop codons, whereas the two remaining *ompK36* mutants had frameshifts due to nucleotide deletion (Table 2). We quantified the expression of the *ompK35* and *ompK36* genes, except for the mutants for which a non-functional porin was expected, based on the detected mutations (Table 2). The mutants obtained from the strains overexpressing the *oqxB* and *acrB* genes were complemented with the wild type *oqxR* and *ramR* genes of strain ATCC13883, respectively (Table 2). We also quantified the expression of the *rarA* and *ramA* genes, encoding positive regulators of the *oqxB* and *acrB* gene, respectively (Table 2), because previous studies suggested that these efflux pump regulators may also control the expression of the porin-encoding genes (De Majumdar et al., 2013, 2015). The expression of the *ompK35* gene was significantly decreased, whereas that of the *rarA* gene was significantly increased in strain KPBj1 E+ and derivatives KPBj1 E+ P-, KPBj1 E+ Δ*acrB* P-, and KPBj1 E+ *ramR*A22V P-, (Table 2). The complementation of these strains with the wildtype *oqxR* gene resulted in normal expression of both the *ompK35* and *rarA* genes, except for strain KPBj1 E+ *ramR*A22V P-, in which OqxAB and AcrAB and the *ramA* gene were overexpressed (Table 2). The expression of the *ompK35* gene was decreased, whereas that of the *ramA* gene was increased in strain KPBj1 M3 Lev and its derivative KPBj1 M3 Lev P-, which are devoid of *rarA* and *oqxRAB* genes and overproduce AcrAB. Deletion of the *ramA* gene from strain KPBj1 M3 Lev and complementation of strain KPBj1 M3 Lev P- with the wildtype *ramR* gene resulted in normal expression of the *ompK35* gene (Table 2). The decreased *ompK35* expression found in strain KPBj1 Rev Δ*acrB* P-, in the presence of normal *ramA* gene expression (Table 2), suggested the involvement of RamA-unrelated control of *ompK35* expression. Therefore, we analyzed the nucleotide sequence (233 bp) upstream of the start codon of the *ompK35* gene. This sequence was identical to that of the parental strain, suggesting that a mechanism other than modification of the promoter region is involved in the decreased expression of the *ompK35* gene. The single mutant selected under ertapenem pressure with a mutation in the *ompK35* but not *ompK36* gene, strain KPBj1 Rev P-, showed decreased expression of the *ompK36* gene. Analysis of the promoter region of the *ompK36* gene showed the presence of a 1196 bp IS element 64 bp upstream of the *ompK36* start codon. This IS element was identified as IS*Kp26* that belongs to the IS*5* family.

**Table 2:** Porin alteration in isogenic strains of *Klebsiella pneumoniae* selected under ertapenem pressure and effect on cefoxitin and ertapenem MICs.

**Strain**	***ompK35***		***ompK36***		Expression (fold change)^*^		**MIC (mg/L)**	
	**sequence**	Expression (fold change)^*^	**sequence**	expression (fold change)^*^	***rarA***	***ramA***	FOX (≤ 8, > 16)^**^	ERT (≤ 0.5, > 1)^**^
ATCC13883	–	–	–	–	–	–	4	0.016
KPBj1 E+	A	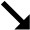 (0.25 ± 0.07)	B	1.41	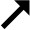 (4087 ± 393)	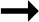 (2.25 ± 0.59)	16	0.016
KPBj1 E+ P-	A	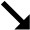 (0.53 ± 0.12)	Frameshift (10-nt deletion)	ND	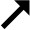 (3325 ± 659)	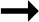 (4.29 ± 2.22)	32	0.5
KPBj1 E+ P- T_*oqxR*−ATCC_	A	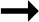 (1.19 ± 0.32)	Frameshift (10-nt deletion)	ND	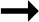 (4.24 ± 0.61)	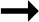 (2.73 ± 1.8)	8 (4)^a^	0.5 (0.016)^a^
KPBj1 E+ Δ*acrB* P-	A	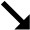 (0.30 ± 0.06)	102 stop codon	ND	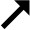 (2787 ± 964)	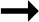 (3.38 ± 1.55)	16 (4)	0.25 (0.016)
KPBj1 E+Δ*acrB* P- T_*oqxR*−ATCC_	A	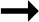 (1.44 ± 0.74)	102 stop codon	ND	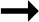 (5.99 ± 1.03)	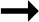 (3.67 ± 2.36)	4 (2)	0.125 (0.016)
KPBj1 E+ *ramR*A22V P-	A	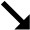 (0.12 ± 0.03)	23 stop codon	ND	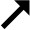 (3448 ± 997)	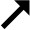 (12.48 ± 0.35)	128 (128)	1 (0.125)
KPBj1 E+ *ramR*A22V P- T_*oqxR*−ATCC_	A	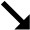 (0.57 ± 0.24)	23 stop codon	ND	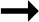 (4.21 ± 0.55)	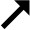 (18.57 ± 6.19)	128 (64)	2 (0.25)
KPBj1 Rev	A	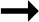 (1.31 ± 0.86)	B	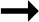 (1.20 ± 0.27)	–	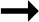 (5.27 ± 1.41)	4	0.016
KPBj1 Rev P-	121 stop codon	ND	B	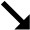 (0.05 ± 0.01)	–	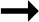 (6.46 ± 1.11)	32	0.25
KPBj1 Rev Δ*acrB*	A	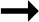 (1.89 ± 0.73)	B	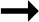 (2.78 ± 0.27)	–	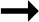 (8.4 ± 1.30)	2	0.016
KPBj1 Rev Δ*acrB* P-	A	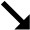 (0.45 ± 0.11)	326–327 deletion	ND	–	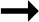 (5.09 ± 0.96)	2	0.125
KPBj1 M3 Lev	A	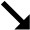 (0.36 ± 0.05)	B	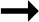 (1.43 ± 0.07)	–	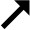 (22.46 ± 18.56)	64	0.03
KPBj1 M3 Lev Δ*ramA*	A	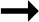 (1.04 ± 0.29)	B	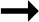 (1.114 ± 0.64)	–	0	4	0.03
KPBj1 M3 Lev P-	A	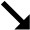 (0.16 ± 0.01)	318 stop codon	ND	–	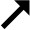 (34.8 ± 14.0)	128	1
KPBj1 M3 Lev P- T_*ramR*−ATCC_	A	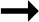 (2.81 ± 0.68)	318 stop codon	ND	–	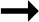 (4.41 ± 0.12)	8 (4)	0.25 (0.03)
KPBj1 M3 Lev Δ*acrB* P-	A	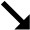 (0.36 ± 0.09)	Frameshift (1-nt deletion) + 102 stop codon	ND	–	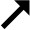 (39.7 ± 1.9)	2 (1)	0.5 (0.03)

* *qRTPCR with strain ATCC13883 as calibrator; P-, porin alteration; nt, nucleotide; ND, not determined; –, absence of the gene; ^a^MIC value in parental strain in parentheses; FOX, cefoxitin; ERT, ertapenem. Arrows indicate the level of gene expression: 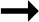, normal; 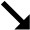, decreased; 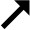, increased; 0, undetectable*.

**Figure 1:**
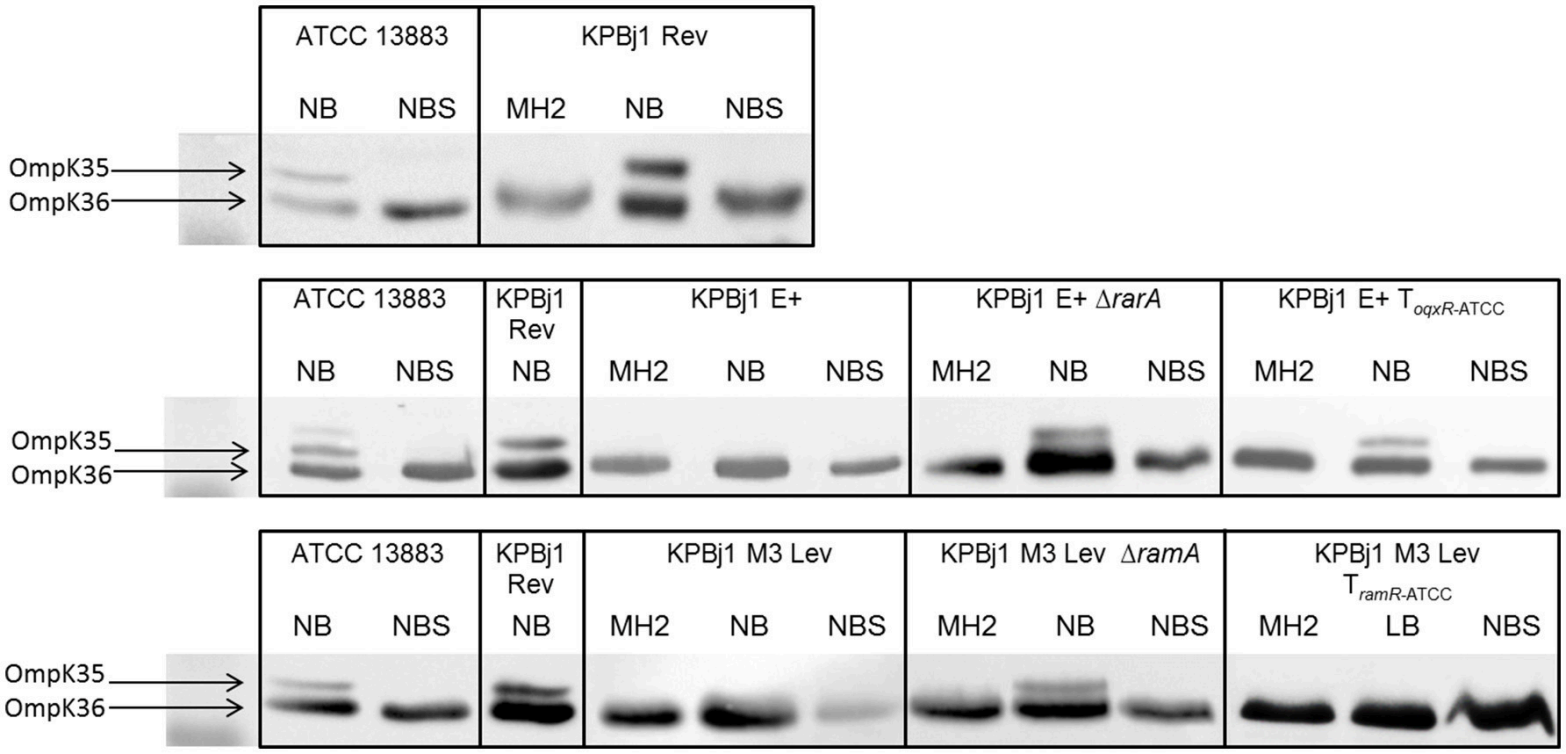
Immunodetection of OmpK35 and OmpK36 synthesis. Detection was performed using polyclonal antibodies directed against denatured OmpC (OmpK36 in *K. pneumoniae*) and OmpF (OmpK35 in *K. pneumoniae*) porins due to cross-recognition. Identical results were obtained for the two porins and the results presented here were obtained using polyclonal antibodies directed against OmpK35. The tested strains were grown in various media: Mueller Hinton II (MH2), nutrient broth (NB), nutrient broth containing sorbitol (NBS), and Luria Broth (LB) when growth in NB was unsuccessful. The figure shows an assembly of the immunodetection signals obtained from different nitrocellulose blots, the black outlines indicating the different blots. It was necessary to prepare several blots to analyse the numerous samples from the various strains grown under various conditions. OmpK35 was detected in strain KPBJ Rev, which contained spontaneous deletions of the *rarA* gene and *oqxABR* operon, and in the strains that we built devoid of the *rarA* gene or which expressed wildtype levels of this gene (KPBjE+T_oqxR*ATCC*_) and in the strain devoid of the *ramA* gene, which were all able to grow in NB. OmpK35 was not detected in strain KPBj Lev M3 T_*ramR*ATCC_, which expresses the *ramA* gene at wildtype levels (see Table 2), because this strain was unable to grow in NB and was subsequently grown in LB.

**Figure 2:**
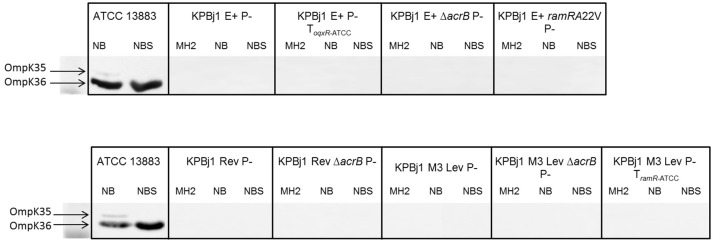
Immunodetection of OmpK35 and OmpK36 synthesis. Immunodetection was performed using polyclonal antibodies directed against the denatured OmpC (OmpK36 in *K. pneumoniae*) OmpF (OmpK35 in *K. pneumoniae*) porins. Identical results were obtained for the two porins. The results presented here are those obtained with polyclonal antibodies directed against OmpK35. The tested strains, which comprise the seven mutants selected under ertapenem and two complemented with the wild type *oqxR* and *ramR* genes of strain ATCCA13883, were grown in various media: Mueller Hinton II (MH2), nutrient broth (NB), or nutrient broth containing sorbitol (NBS). The figure shows an assembly of the immunodetection signals obtained from different nitrocellulose blots, the black outlines indicating the different blots. It was necessary to prepare several blots to analyse the numerous samples from the various strains grown under various conditions. Neither OmpK36 nor OmpK35 were detected in any tested strain.

We performed porin immunodetection experiments on several parental strains and their derivatives selected under ertapenem. OmpK35 was detected in parental strains that could grow on NB broth with normal expression of the *ramA* gene and deletion of the *oqxABR* and *rarA* genes (strain KPBj1 Rev), a *rarA* gene knockout, a complementation with the wildtype *oqxR* gene, and a *ramA* gene knockout. We were unable to perform this experiment for the strain complemented with the wildtype *ramR* gene because of its inability to grow in NS broth (Figure 1). Neither OmpK36 nor OmpK35 were detectable in the strains obtained under the selective pressure of ertapenem (Figure 2).

### Effect of combined porin loss and varying efflux pump production on the activity of various antibiotics

#### Cefoxitin and ertapenem

We previously showed that porin alteration impaired the activity of cefoxitin and not that of nalidixic acid and chloramphenicol (Bialek et al., 2010). Thus, we investigated the effect of porin loss, alone and associated with varying levels of efflux pump production, on the susceptibility to cefoxitin (Table 2) and ertapenem, used to select porin-loss variants (Tables 2, 3). The lowest MICs of both cefoxitin and ertapenem, i.e., 2 and 0.0016 mg/L, respectively, were observed in strain KPBj1 Rev Δ*acrB*, devoid of the AcrAB and OqxAB pumps. The MIC of cefoxitin was unchanged in the porin-loss derivative of this strain, whereas that of ertapenem was significantly higher (0.125 mg/L). This was also true for strain KPBj1 M3 Lev Δ*acrB*, devoid of AcrAB and OqxAB, and its porin-loss derivative, as well as strain KPBj1 M3 Lev, which overproduced the AcrAB pump, and its porin-loss derivative. Overall, these results suggest that OmpK35 and OmpK36 alteration impairs ertapenem activity and not that of cefoxitin. However, the cefoxitin MIC significantly increased in two parental strain/porin-loss derivative couples (4 vs. 32 mg/L in KPBj1 Rev/KPBj1 Rev P- and 4 vs. 16 mg/L in KPBj1 E+ Δ*acrB*/KPBj1 E+ Δ*acrB* P-). We showed above that cefoxitin is slightly expelled from the parental strain of these two couples associated the AcrAB pump normally produced in strain KPBj1 Rev and the overproduction of OqxAB in strain KPBj1 E+ Δ*acrB*. Thus, the increase in the cefoxitin MIC in the porin-loss derivatives of these two strains may be due to a decrease in the periplasmic concentration of cefoxitin caused by a slight efflux and decreased influx due to porin loss. We did not observe the effect of such reduced influx of cefoxitin in the porin-loss derivatives of the strains that overproduce the AcrAB pump. In this context, efflux had a higher impact on cefoxitin accumulation than influx. For ertapenem, the activity is systematically affected by porin loss and when the AcrAB pump is overexpressed.

**Table 3:** MICs of 9 β-lactams, tigecycline, and colistin in isogenic *Klebsiella pneumoniae* strains with and without overproduction of efflux pumps and porin alterations.

**Strain (gene expression)**	**CMI mg/L**										
	ERT ≤ 0.5, >1^*^	**IMI ≤ 2, >8**	**PIP ≤ 8, >16**	**PIP+TAZ ≤ 8, >16**	**CFO ≤ 1, >1**	**CFO+TAZ ≤ 1, >1**	**CAZ ≤ 1, >4**	**CAZ+AVI ≤ 8, >8**	**TEM ≤ 8, >8**	**TIG ≤ 1, >2**	**COL ≤ 2, >2**
ATCC13883	0.016	1	16	8	0.5	0.25	0.5	0.25	4	0.5	2
KPBj1 E+ T_oqxR_^*^ ( 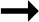 *acrB*, 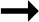 *oqxB*)	0.016	0.5	>128	64	0.5	0.25	0.25	0.25	4	1	1
KPBj1 E+ P- T_oqxR_^**^	0.5	0.25	>128	128	0.5	0.25	0.5	0.5	2	1	0.5
KPBj1 E+ Δ_*acrB*_ T_oqxR_^**^ (0 *acrB*, 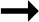 *oqxB*)	0.016	0.25	4	≤ 0.25	1	0.5	0.25	0.125	4	0.5	0.5
KPBj1 E+Δ_*acrB*_ P- T_oqxR_^***^	0.125	0.5	4	0.5	1	0.5	0.125	0.25	4	0.5	0.25
KPBj1 Rev ( 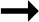 *acrB*, 0 *oqxB*)	0.016	0.25	8	4	0.5	0.25	0.25	0.125	4	1	2
KPBj1 Rev P-	0.25	0.25	8	4	1	0.25	0.25	0.25	4	1	8
KPBj1 Rev Δ*_*acrB*_* (0 *acrB*, 0 *oqxB*)	0.016	0.25	4	≤ 0.25	0.5	0.25	0.25	0.125	4	0.5	4
KPBj1 Rev Δ*_*acrB*_* P-	0.125	0.5	8	≤ 0.25	1	0.25	0.25	0.25	8	0.5	2
KPBj1 E+ ( 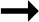 *acrB*, 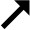 *oqxB*)	0.016	0.25	8	8	0.5	0.5	0.5	0.25	4	2	2
KPBj1 E+ P-	0.5	0.25	8	8	0.5	0.25	0.25	0.125	4	2	2
KPBj1 E+ Δ*_*acrB*_* (0 *acrB*, 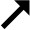 *oqxB*)	0.016	0.25	4	1	0.5	0.25	0.25	0.125	4	0.5	2
KPBj1 E+ Δ*_*acrB*_* P-	0.25	0.5	8	1	0.5	0.25	0.125	0.125	4	0.5	2
KPBj1 M3 Lev ( 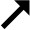 *acrB*, 0 *oqxB*)	0.03	0.125	16	16	2	0.5	1	0.5	8	4	2
KPBj1 M3 Lev P-	1	0.125	32	8	1	0.5	0.5	0.25	8	4	2
KPBj1 E+ *ramR*A22V T_oqxR_^**^( 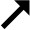 *acrB*, 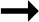 *oqxB*)	0.25	0.125	>128	128	1	0.25	0.5	0.25	8	8	1
KPBj1 E+ *ramR*A22V P- T_oqxR_^**^	2	0.25	>128	128	1	0.5	0.5	0.5	8	8	0.5
KPBj1 E+ *ramR*A22V ( 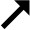 *acrB*, 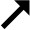 *oqxB*)	0.125	0.125	16	8	2	0.5	1	0.5	8	8	2
KPBj1 E+ *ramR*A22V P-	1	0.125	16	16	1	0.5	0.5	0.5	8	8	1

ERT, ertapenem; IMI, imipenem; PIP, piperacillin; PIP+TAZ, piperacillin+tazobactam; CFO, ceftolozane; CFO+TAZ, ceftolozane+tazobactam; CAZ, ceftazidime; CAZ+AVI, ceftazidime+avibactam; TEM, temocillin; TIG, tigecycline; COL, colistin; P-, porin alteration;

* breakpoints; 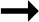, normal gene expression; 0: undetectable gene expression; 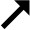, increased ex gene expression;

** pSC-A plasmid vector encoding resistance to penicillins;

*** *pBBRMCS-III plasmid vector encoding resistance to tetracyclines*.

#### Antibiotics recommended to treat infections due to multidrug-resistant *K. pneumoniae*

Although cefoxitin and ertapenem are not hydrolysed by class A β-lactamases, including ESBL (Richmond, 1978), these antibiotics are rarely used to treat infections caused by ESBL-producing *K. pneumoniae* clinical isolates. Indeed, treatment failures due to *in-vivo* selection of porin-loss strains resistant to these antibiotics have been described for ESBL-producing *K. pneumoniae* clinical isolates (Pangon et al., 1989; Elliott et al., 2006; Skurnik et al., 2010). Imipenem and meropenem are currently used for the treatment of these infections. However, the emergence of plasmid-mediated carbapenemases highlights the need for molecules other than carbapenems to treat such infections (Perez et al., 2016; Tamma and Rodriguez-Bano, 2017). In this context, temocillin and β-lactams combined with β-lactamase inhibitors, namely pipiracillin+tazobactam, ceftolozane+tazobactam, or ceftazidime+avibactam, have been suggested. We determined the effect of porin loss, alone and associated with various levels of efflux pump production, on the activity of the cited β-lactams, as well as on that of tigecycline and colistin (Table 3), as these antibiotics are sometimes used when resistance extends to all β-lactams (Cobo et al., 2008; Giamarellou and Poulakou, 2009).

Imipenem MICs varied from 0.125 to 0.5 mg/L (Table 3). The imipenem MIC observed when the AcrAB pump, OqxAB pump, or both were either not produced or overproduced did not show significant differences (0.25 mg/L) from the value observed when the two pumps were normally produced (0.5 mg/L) (Table 3). This suggests that imipenem is not a good substrate of the two pumps. On the other hand, unlike ertapenem, the imipenem MICs were not significantly increased in any porin-loss derivative (Table 3).

Piperacillin MICs varied from 4 to 32 mg/L (Table 3), except for the *K. pneumoniae* strains transformed by the pSC-A plasmid vector (Table S1), which harbours a gene encoding a class A β-lactamase that can hydrolyse penicillin molecules. Therefore, the results obtained for these transformed strains for piperacillin alone and when associated with tazobactam, a class A β-lactamase inhibitor, were uninterpretable. In the other strains, the piperacillin MICs were 4–8 mg/L in those with or without porins and with either no expression of the two pumps or wildtype expression of one of the two pumps, suggesting that piperacillin is not expelled by the two pumps when normally produced. Piperacillin MICs were >8 mg/L, superior to the low breakpoint, for strains with or without porins that overproduce the AcrAB pump, indicating that piperacillin is a substrate of the overproduced AcrAB pump. On the other hand, these results show that porin alteration has no significant impact on piperacillin activity. The addition of 4 mg/L tazobactam resulted in significant piperacillin+tazobactam MIC decreases (from 4- to 32-fold) only in strains with the *acrB* gene knockout (Table 3), strongly suggesting that tazobactam is expelled by the normally produced AcrAB pump. The subsequently higher concentration of tazobactam in the periplasmic space of the strains with a deleted *acrB* gene should increase inhibition of the chromosomal class A β-lactamase of *K. pneumoniae* and thus increase piperacillin activity. However, these results show that porin loss did not change the activity of piperacillin-tazobactam.

Ceftolozane MICs varied from 0.5 to 1 mg/L (Table 3) in all the strains, except KPBj1 M3 Lev and KPBj1 E+ *ramR*A22V, which overproduce the AcrAB pump (MIC: 2 mg/L). These findings suggest that normal pump production, overexpression of the OqxAB pump, and porin loss do not affect ceftolozane activity. Inversely, decreases in ceftolozane MICs in two of the three strains overproducing AcrAB suggest that ceftolozane is a substrate of this pump. The addition of 4 mg/L of tazobactam resulted in a 4-fold decrease of the ceftolozane+tazobactam MIC in all strains overproducing the AcrAB pump.

Ceftazidime MICs varied from 0.125 to 1 mg/L (Table 3) and there was no significant difference between the parental strains and their derivatives devoid of the *acrB* and/or *oqxB* genes and OmpK35 and OmpK36. These results suggest that normal production of the two pumps and the absence of OmpK35 and OmpK36 do not affect ceftazidime activity. The highest ceftazidime MIC (1 mg/L) observed in two strains that overexpressed the AcrAB pump suggests that tceftazidime activity may be affected by such overproduction. The addition of avibactam, a non-β-lactam inhibitor of classes A, C, and D β-lactamases, did not alter the MIC values.

Temocillin MICs, which varied from 2 to 8 (Table 3), displayed no significant difference between parental strains and their derivatives devoid of either porins or efflux pumps, and their derivatives overproducing one or two of the pumps. These results suggest that temocillin is not a substrate of the two pumps and the absence of OmpK35 and OmpK36 does not affect its activity.

Concerning non β-lactam antibiotics (Table 3), neither porin loss nor *oqxB* gene overexpression affected tigecycline activity. Conversely, increased *acrB* expression resulted in significant increases in the MIC of tigecycline. Colistin MICs varied from 0.25 to 8 mg/L, with a modal MIC of 2 mg/L. The two strains in which the colistin MICs were 4 and 8 mg/L, superior to the breakpoint, were the *acrB*-loss and porin-loss derivatives of strain KPBj1 Rev, respectively. The first derivative is devoid of the two efflux pumps, whereas the second is devoid of the two porins.

### The effect of combining varying efflux pump production and porin alteration on the β-lactam susceptibility of DHA-1, CTX-M-15, or OXA-48-producing isogenic *K. pneumoniae* strains

We evaluated the ertapenem, imipenem, piperacillin, piperacillin+tazobactam, ceftolozane, ceftolozane+tazobactam, ceftazidime, ceftazidime+avibactam, and temocillin susceptibility of isogenic strains transformed with plasmids encoding the most commonly identified β-lactamases of AmpC type, i.e., DHA-1, ESBL type, i.e., CTX-M-15 (here associated with OXA-1 and TEM-1 production), and carbapenemase type, i.e., OXA-48-like, in European *K. pneumoniae* isolates (Baraniak et al., 2013; Potron et al., 2013; Freitas et al., 2014; Rodrigues et al., 2014; Ruiz-Garbajosa et al., 2016) in terms of clinical categorization (susceptible: S, intermediate susceptible: I, and resistant: R). The isogenic strains used were selected to evaluate how the overproduction of each or both pumps, alone or associated with porin loss, modulate the β-lactam susceptibility of β-lactamase-producing strains, i.e., category S changed to category I or R and *vice versa*. Transformation of the strain producing normal levels of both AcrAB and OqxAB pumps (KPBj1 E+ T_*oqxR*_) with the plasmids encoding DHA-1, CTX-M-15, or OXA-48 failed, probably because of the presence of the plasmid used for complementation experiments. Therefore, we used the vector plasmid-free strain producing normal levels of the AcrAB pump and devoid of the OqxAB pump (KPBj1 Rev), as the β-lactam MICs were identical in the two strains, except for piperacillin (alone and associated with tazobactam), which was hydrolysed by the penicillinase harbored by the *oqxR* transfer vector in strain KPBj1 E+ T_*oqxR*_ (Table 3). Overall, we studied the same eight isogenic strains producing either DHA-1, CTX-M-15, or OXA-48 (Table 4).

**Table 4:** Effect of efflux pump overexpression alone and associated with porin alterations on the β-lactam susceptibility of isogenic strains of *K. pneumoniae* producing DHA-1, CTX-M-15, and OXA-48.

**Strain (*acrB* and *oqxB* gene expression)**	**CMI mg/L (susceptibility categorization)**								
	ERT ≤ 0.5, >1^*^	**IMI ≤ 2, >8**	**PIP ≤ 8, >16**	**PIP+TAZ ≤ 8, >16**	**CFO ≤ 1, >1**	**CFO+TAZ ≤ 1, >1**	**CAZ ≤ 1, >4**	**CAZ+AVI ≤ 8, >8**	**TEM ≤ 8, >8**
KPBj 1 Rev ( 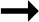 *acrB*, 0 *oqxB*) DHA-1	0.25 (S)	0.25 (S)	>128 (R)	>128 (R)	32 (R)	8 (R)	128 (R)	2 (S)	4 (S)
KPBj 1 Rev P- DHA-1	>16 (R)	2 (S)	>128 (R)	>128 (R)	>64 (R)	>64 (R)	>128 (R)	8 (S)	8 (S)
KPBj1 E+ ( 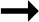 *acrB*, 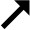 *oqxB*) DHA−1	1 (I)	0.25 (S)	>128 (R)	>128 (R)	64 (R)	16 (R)	>128 (R)	4 (S)	8 (S)
KPBj1 E+ P- DHA-1	>16 (R)	8 (I)	>128 (R)	>128 (R)	32 (R)	8 (R)	128 (R)	4 (S)	4 (S)
KPBj1 M3 Lev ( 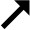 *acrB*, 0 *oqxB*) DHA-1	1 (I)	0.125 (S)	>128 (R)	>128 (R)	64 (R)	32 (R)	>128 (R)	8 (S)	16 (R)
KPBj1 M3 Lev P- DHA-1	>16 (R)	8 (I)	>128 (R)	>128 (R)	64 (R)	32 (R)	>128 (R)	8 (S)	4 (S)
KPBj1 E+ *ramR*A22V ( 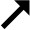 *acrB*, 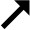 *oqxB*) DHA-1	16 (R)	0.5 (S)	>128 (R)	>128 (R)	>64 (R)	64 (R)	>128 (R)	8 (S)	16 (R)
KPBj1 E+ *ramR*A22V P- DHA-1	>16 (R)	4 (I)	>128 (R)	>128 (R)	64 (R)	64 (R)	>128 (R)	16 (R)	16 (R)
KPBj 1 Rev ( 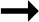 *acrB*, 0 *oqxB*) CTX-M-15	0.125 (S)	0.125 (S)	>128 (R)	16 (I)	64 (R)	1 (S)	32 (R)	0.5 (S)	8 (S)
KPBj 1 Rev P- CTX-M-15	8 (R)	0.5 (S)	>128 (R)	128 (R)	>64 (R)	16 (R)	64 (R)	1 (S)	16 (R)
KPBj1 E+ ( 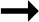 *acrB*, 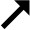 *oqxB*) CTX-M-15	0.25 (S)	0.125 (S)	>128 (R)	32 (R)	>64 (R)	2 (R)	64 (R)	0.5 (S)	8 (S)
KPBj1 E+ P- CTX-M-15	8 (R)	0.5 (S)	>128 (R)	64 (R)	>64 (R)	16 (R)	64 (R)	0.5 (S)	8 (S)
KPBj1 M3 Lev ( 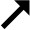 *acrB*, 0 *oqxB*) CTX-M-15	0.125 (S)	0.0625 (S)	>128 (R)	8 (S)	>64 (R)	1 (S)	64 (R)	1 (S)	16 (R)
KPBj1 M3 Lev P- CTX-M-15	8 (R)	0.125 (S)	>128 (R)	8 (S)	>64 (R)	1 (S)	32 (R)	1 (S)	8 (S)
KPBj1 E+ *ramR*A22V ( 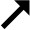 *acrB*, 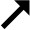 *oqxB*) CTX-M-15	0.25 (S)	0.0625 (S)	>128 (R)	16 (I)	>64 (R)	1 (S)	32 (R)	0.5 (S)	16 (R)
KPBj1 E+ *ramR*A22V P- CTX-M-15	8 (R)	0.25 (S)	>128 (R)	32 (R)	>64 (R)	4 (R)	32 (R)	0.5 (S)	16 (R)
KPBj 1 Rev ( 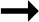 *acrB*, 0 *oqxB*) OXA-48	2 (R)	2 (S)	>128 (R)	>128 (R)	2 (R)	0.5 (S)	0.25 (S)	0.5 (S)	512 (R)
KPBj 1 Rev P- OXA-48	>16 (R)	>64 (R)	>128 (R)	>128 (R)	8 (R)	1 (S)	0.5 (S)	0.5 (S)	>1024 (R)
KPBj1 E+ ( 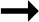 *acrB*, 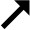 *oqxB*) OXA-48	8 (R)	2 (S)	>128 (R)	>128 (R)	2 (R)	1 (S)	0.5 (S)	0.5 (S)	1024 (R)
KPBj1 E+ P- OXA-48	>16 (R)	64 (R)	128 (R)	128 (R)	2 (R)	0.5 (S)	0.125 (S)	0.25 (S)	512 (R)
KPBj1 M3 Lev ( 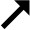 *acrB*, 0 *oqxB*) OXA-48	>16 (R)	2 (S)	>128 (R)	>128 (R)	4 (R)	1 (S)	1 (S)	1 (S)	>1024 (R)
KPBj1 M3 Lev P- OXA-48	>16 (R)	>64 (R)	>128 (R)	>128 (R)	2 (R)	1 (S)	0.5 (S)	1 (S)	>1024 (R)
KPBj1 E+ *ramR*A22V ( 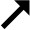 *acrB*, 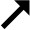 *oqxB*) OXA-48	>16 (R)	4 (I)	>128 (R)	>128 (R)	2 (R)	1 (S)	1 (S)	0.5 (S)	1024 (R)
KPBj1 E+ *ramR*A22V P- OXA-48	>16 (R)	64 (R)	>128 (R)	>128 (R)	4 (R)	1 (S)	1 (S)	0.5 (S)	1024 (R)

ERT, ertapenem; IMI, imipenem; PIP, piperacillin; PIP+TAZ, piperacillin+tazobactam; CFO, ceftolozane; CFO+TAZ, ceftolozane+tazobactam; CAZ, ceftazidime; CAZ+AVI, ceftazidime+avibactam; TEM, temocillin; P-, porin alteration;

* *breakpoints; 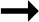, normal gene expression; 0 : undetectable gene expression; 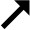, increased gene expression; S, susceptible; I, intermediate susceptible; R, resistant*.

For the eight strains producing DHA-1 (Table 4), the strain producing normal levels of the AcrAB pump was S to ertapenem, imipenem, ceftazidime+avibactam, and temocillin and R to piperacillin, piperacillin+tazobactam, ceftolozane, ceftolozane+tazobactam, and ceftazidime. Overproduction of the pumps only modified the susceptibility profile for ertapenem and temocillin. Thus, strains overproducing one of the two pumps became I to ertapenem, whereas that overproducing the two pumps became R. Strains overproducing AcrAB became R to temocillin. Porin alteration significantly modified the susceptibility profile of some parental strains but only for ertapenem, imipenem, and ceftazidime+avibactam. Thus, the porin-loss derivative of the strain producing normal levels of the AcrAB pump became R to ertapenem, the porin-loss derivatives of the strains overproducing at least one pump became I to imipenem, and the porin-loss derivative of the strain overproducing the two pumps became R to ceftazidime+avibactam.

For the eight strains producing CTX-M-15 (Table 4), the strain producing normal levels of AcrAB was S to ertapenem, imipenem, ceftolozane+tazobactam, ceftazidime+avibactam, and temocillin, I to piperacillin+tazobactam, and R to piperacillin, ceftolozane, and ceftazidime. Pump overproduction slightly modified this susceptibility profile. The OqxAB-overproducing strain became R to ceftolozone+tazobactam, with a MIC of 2 mg/L. The strains overproducing the AcrAB pump or the two pumps became R to temocillin. Inversely, the strain overproducing the AcrAB pump (KPBj1 M3 Lev CTX-M-15) became S to piperacillin+tazobactam (MIC = 8 vs. 16 mg/L in the strain producing normal levels of the AcrAB pump). Porin alteration did not modify the susceptibility of the parental strains for imipenem, piperacillin, piperacillin+tazobactam ceftolozane, ceftazidime, or ceftazidime+avibactam. All the porin-loss derivatives were R to ertapenem, independently of the level of pump production. All porin-loss derivatives became R to ceftolozane+tazobactam, except for the porin-loss derivative of strain KPBj1 M3 Lev CTX-M-15. The porin-loss derivatives of the strain producing normal levels of AcrB became R to temocillin, whereas that of the strain overproducing this pump became S.

For the eight strains producing OXA-48 (Table 4), the strain producing normal levels of AcrAB was S to imipenem, ceftolozane+tazobactam, ceftatazidime, and ceftazidime+avibactam, and R to ertapenem, piperacillin, piperacillin+tazobactam, ceftolozane, and temocillin. Pump overproduction did not modify this susceptibility profile, except for the strain overproducing the two pumps, which became I to imipenem. Porin alteration significantly modified the susceptibility profile of the parental strains for only imipenem. Thus, all porin-loss derivatives became R to imipenem independently of the level of pump production.

## Discussion

In addition to the diversity of β-lactamases involved in the acquired resistance to β-lactams in *K. pneumoniae* clinical isolates, membrane permeability alterations have also been observed in isolates resistant to multiple antibiotics (Hasdemir et al., 2004; Dahmen et al., 2012; López-Camacho et al., 2014). Here, we developed a protocol including 50 isogenic strains designed to elucidate the contribution of various membrane permeability alterations, as well as their association with resistance to a large panel of antibiotics.

The part of the study focusing on various efflux producers showed that AcrAB contributes to the intrinsic resistance to nalidixic acid, chloramphenicol, and cefoxitin, as previously described in *Escherichia coli* (Mazzariol et al., 2000; Sulavik et al., 2001). It also provided new findings: (i) the contribution of OqxAB to the intrinsic resistance of *K. pneumoniae* to chloramphenicol, (ii) the production of OqxAB at different levels, including an intermediate level when the transcriptional regulator RarA is absent, (iii) the relationship between the level of production of OqxAB and the MIC values of the three antibiotics, and (v) the absence of an intermediate level of AcrAB production when the transcriptional regulator RamA is deleted. The analyses of the antibiotics of interest for treating infections due to multidrug-resistant *K. pneumoniae* (ertapenem, imipenem, piperacillin±tazobactam, ceftolozane±tazobactam, ceftazidime±avibactam, temocillin, tigecycline, and colistin), showed that all, but piperacillin+tazobactam, are not expelled by one or the other pump when they produced at normal levels. In the absence of normal AcrAB efflux, the piperacillin+tazobactam MIC decreased considerably. This finding explains the lower piperacillin+tazobactam MIC previously observed in *K. pneumoniae* strains when grown in the presence of efflux pump inhibitors (Pagès et al., 2009). Our findings are in accordance with the results of Mazzariol et al. who assessed the impact of AcrAB deletion on various β-lactams, except for piperacillin (Mazzariol et al., 2000). Indeed, they and also Opperman et al. (Opperman et al., 2014) who tested a new efflux pump inhibitor, found that AcrAB contributes to the intrinsic resistance of *E. coli* to piperacillin, whereas we found that this was not the case for *K. pneumoniae*. The discrepancy between the two species is likely due to the hydrolysis rate of piperacillin by the class A chromosomal β-lactamase of *K. pneumoniae*, which can mask the eventual efflux of piperacillin by AcrAB produced at normal levels. Deletion of the chromosomal β-lactamase of *K. pneumoniae* should allow clarification of whether piperacillin is expelled by pumps produced at normal levels. We found that AcrAB overproduction affects not only the activity of tigecycline, as previously described (Bialek-Davenet et al., 2015; Fang et al., 2016), but also that of ertapenem, piperacillin, ceftolozane, and temocillin. We also found that the MICs of ceftolozane+tazobactam were 4-fold lower than ceftolozane MICs in strains overproducing AcrAB. These decreases of the MICs may be related to the competitive expulsion of ceftolozane and tazobactam by the same pump resulting in an increase in the ceftolozane concentration in the bacterial cell. Overall, these results show that the activity of various antibiotics is differentially affected by identical efflux changes in *K. pneumoniae*, possibly due to their respective affinity for pump transporters.

The alteration of permeability related to porin loss was obtained not by the knockout procedure of the genes encoding the OmpK35 and OmpK36 porins, but through ertapenem selection pressure, as described in the clinic (Elliott et al., 2006; Skurnik et al., 2010). All derivatives had a non-functional OmpK36 due to various gene mutations among which some were reported in clinical isolates (Hernández-Allés et al., 1999; Cuzon et al., 2010). The mutant with the defective *ompK36* promoter was also the only mutant with a non-functional OmpK35. The other mutants displayed varying levels of OmpK35 production, which we molecularly and phenotypically showed to be linked to the level of production of the transcriptional regulators RarA and RamA in all but one case. As expected, none of the non-functional OmpK36 molecules were immunodetected. This was also true for OmpK35, independently of its protein structure and level of production. This suggests, at least for the strains in which the *ompK35* gene was normally transcribed, the occurrence of post-translational regulation processes, as previously reported for *Escherichia coli* strains exposed to antibiotics (Viveiros et al., 2007). We found that the absence of porins varyingly affected the antibacterial activity of β-lactams tested in the absence of plasmid-mediated β-lactamases: there was (i) never a significant increase in the MIC over that of the parental strain for imipenem, piperacillin, piperacillin+tazobactam, ceftolozane, ceftolozane+tazobactam, or temocillin, independently of efflux pump production, (ii) always a significant increase in the MIC for ertapenem, independently of efflux pump production, and (iii) a significant increase in the MIC for cefoxitin, only when porin alteration was associated with the slight efflux of cefoxitin. We observed an original phenomenon for ceftolozane+tazobactam relative to ceftolozane, namely a 4-fold decrease in the MIC of ceftolozane+tazobactam in strains KPBj1 Rev P- and KPBj1 Rev Δ_*acrB*_ P-. We found that these strains display specific features of the *ompK35* gene relative to the other porin-loss derivatives: a mutation in the coding region of the gene in the first strain, and downregulation of gene expression by a transcriptional regulator other than RamA in the second. If these features affect the activity of the ceftolozane+tazobactam combination, it likely concerns the activity of tazobactam, as we observed decreased ceftolozane MICs when ceftolozane was combined with tazobactam. Finally, we observed colistin resistance in two strains, one devoid of the two efflux pumps and one devoid of the two porins. Further analyses are required to investigate the possible relationship between the alteration of membrane structure and the modification of colistin activity.

The last aspect of the protocol addresses the transfer of various β-lactamases into the isogenic strains overproducing efflux pumps alone or in association with porin absence. In this context, we showed susceptibility of ertapenem, in decreasing order, to the production of the β-lactamases OXA-48, DHA-1, and CTX-M-15, with a major effect of porin loss on strains producing CTX-M-15 to render them resistant to ertapenem. For imipenem, our study clarified the role of the overproduction of efflux pumps to render porin-devoid, DHA-1-producing *K. pneumoniae* strains I to imipenem, as previously suggested in clinical isolates (Shi et al., 2013), and also the crucial role of porin loss to render OXA-48-producing *K pneumoniae* strains R to imipenem, as previously suggested in clinical isolates (Lunha et al., 2016).

Concerning ceftolozane, we found that our strains producing DHA-1 and CTX-M-15 were R to this antibiotic independently of porin production and efflux level, as previously published (Titelman et al., 2011), However, we found for the first time that it was also the case of our strains producing OXA-48 in which the ceftolozane activity was restored in the presence of 4 mg/L tazobactam. Comparing the *in vitro* results that we obtained for ceftolozane+tazobactam in our strains producing various β-lactamases with the few clinical results available thus far, we found a good concordance (Livermore et al., 2017).

As expected, our strains producing OXA-48 were S to ceftazidime, whereas those producing DHA-1 and CTX-M-15 were R. The addition of 4 mg/L avibactam rendered all ceftazidime-R strains S, except for the strain in which DHA-1 production, overproduction of the two pumps, and porin loss were associated. However, the ceftazidime-avibactam MICs were significantly higher in the S strains producing DHA-1 (2–8 mg/L) than those producing CTX-M-15 (0.5–1 mg/L). This may explain why the strain overproducing the two pumps and devoid of porins was S to ceftazidime-avibactam when it produced CTX-M-15 and R when it produced DHA-1. A comparison of the ceftazidime+avibactam susceptibility of our strains with that of *K. pneumoniae* clinical isolates currently available in the literature showed good concordance with previous reports (Aktaş et al., 2012; Vasoo et al., 2015).

As expected (Woodford et al., 2014), all our strains producing OXA-48 were R to temocillin, with high MICs (512–>1204 mg/L). The highest MIC value observed in nine of the 16 strains producing DHA-1 and CTX-M-15 was16 mg/L. These nine strains were categorized as R to temocillin based on the published CASFM MIC breakpoints that we used (≤8 mg/L–> 8 mg/L). The temocillin MIC values observed in the seven strains categorized as S varied from 4 to 8 mg/L, which were similar to the MICs observed in the isogenic strains that do not produce plasmid-mediated β-lactamases. Thus, the narrow range of temocillin MICs displayed by all tested strains, except those producing OXA-48, strongly suggests that membrane permeability changes and the production of class A and class C β-lactamase slightly affect temocillin activity.

In conclusion, this study clearly shows that the modification of antibiotic transport across bacterial membranes, namely influx and efflux, has various affects depending on the type of change of antibiotic transport, the type of β-lactamases produced, the interplay between the various mechanisms, and the chemical group of antibiotics.

## Author contributions

M-HN-C designed the research. NM and KG performed both bacteriological and molecular biology assays. ED performed immunodetection assays. M-HN-C supervised the overall project with contributions from J-MP for the part focusing on porin regulation and synthesis. M-HN-C wrote the manuscript with contributions from J-MP. All the authors read the manuscript and approved it for submission.

### Conflict of interest statement

The authors declare that the research was conducted in the absence of any commercial or financial relationships that could be construed as a potential conflict of interest.
