# Supplementary material for: Interplay Between Membrane Permeability and Enzymatic Barrier Leads to Antibiotic-Dependent Resistance in Klebsiella Pneumoniae
Source: Front Microbiol. 2018 Jun 29;9:1422. doi: 10.3389/fmicb.2018.01422 (PMC6034560; doi:10.3389/fmicb.2018.01422)
Supplement: Supplementary file 1 [file Table_1.PDF]

INTERPLAY BETWEEN MEMBRANE PERMEABILITY AND ENZYMATIC BARRIER LEADS TO ANTIBIOTIC-DEPENDENT RESISTANCE IN *KLEBSIELLA PNEUMONIAE*

Marie-Hélène Nicolas-Chanoine[1, 2, 3 *], Noémie Mayer[1], Kathleen Guyot[1#], Estelle Dumont[4], Jean-Marie Pagès[4].

[1]Service de Microbiologie, Hôpital Beaujon, AP-HP, Clichy, France, [2]Faculté de Médecine D. Diderot, Paris, France, [3]INSERM UMR 1137, Université Paris 7, Paris, France, [4] UMR_MD1, Aix-Marseille Univ, IRBA, Marseille, France.

Table S1. List of bacterial strains, plasmids and primers used in this study.

Strain, plasmid or primer	Description	Reference or source
**Strains**		
ATCC 13883	Wild-type reference strain	(Skerman et al., 1989)
KPBj1 E+	Clinical isolate with mutation in the gene *oqxR:* overexpression of *oqxAB*, basic expression of *acrAB*	(Bialek-Davenet et al., 2015)
KPBj1 E+ $T_{\textit{oqxR}\text{-ATCC}}$	KPBj1 E+ complemented with plasmid pSC-A-amp/kan-*oqxR*-ATCC: Amp^r, Kan^r: basic expression of *oqxAB* and *acrAB*	(Bialek-Davenet et al., 2015)
KPBj1 E+ Δ*acrB*	*acrB*-deleted strain derived from KPBj1 E+: Kan^r, overexpression of *oqxAB*, no expression of *acrAB*	(Bialek-Davenet et al., 2015)
KPBj1 E+ P-	KPBj1 E+ derivative with porin alteration	(Bialek et al., 2010)
KPBj1 Rev	*oqxRAB*-deleted KPBj1 E+: no expression of *oqxAB*, basic expression of *acrAB*	(Bialek-Davenet et al., 2015)
KPBj1 Rev P-	KPBj1 Rev derivative with porin alteration	(Bialek et al., 2010), this study
KPBj1 M3 Lev	*ramR*-deleted mutant derived from KPBj1 Rev: no expression of *oqxAB*, overexpression of *acrAB*	(Bialek-Davenet et al., 2013)
KPBj1 M3 Lev $T_{\textit{ramR}\text{-ATCC}}$	KPBj1 M3 Lev complemented with plasmid pSC-A-amp/kan-*ramR*-ATCC: Amp^r, Kan^r : no expression of *oqxAB* and basic expression of *acrAB*	(Bialek-Davenet et al., 2013)
**Plasmids**		
pSC-A-amp/kan-*oqxR*-ATCC	pSC-A-amp/kan plasmid vector containing the cloned wild-type *oqxR* gene, Amp^r, Kan^r	(Bialek-Davenet et al., 2015)
pSC-A-amp/kan-*ramR*-ATCC	pSC-A-amp/kan plasmid vector containing the cloned wild-type *ramR* gene, Amp^r, Kan^r	(Bialek-Davenet et al., 2011)
pKOBEG199	pBR322 derivative containing the λ Red region and *araC* gene of pKOBEG, Tet^r	(Balestrino et al., 2005), this study
pBBRMCS-III-*oqxR*-ATCC	pBBRMCS-III plasmid vector containing the cloned wild-type *oqxR* gene, Tet^r	(Kovach et al., 1995), this study
**Primers**		
PCR and Sequencing	Sequence 5'-3'	
$\textit{bla}_{\text{DHA-1}}$	DHA 1U- CACACGGAAGGTTAATTCTGA	(Pai et al., 2004)
	DHA 1L- CGGTTATACGGCTGAACCTG	
$\textit{bla}_{\text{CTX-M15}}$	CTX-C1- ATGTGCAGCACCAGTAAAGT	(Armand-Lefèvre et al., 2003)
	CTX-C2- ACCGCGATATCGTTGGTGG	
$\textit{bla}_{\text{OXA-1}}$	OXA A1- TCAACTTTCAAGATCGCA	(Armand-Lefèvre et al., 2003)
	OXA A2- GTGTGTTTAGAATGGTGA	
$\textit{bla}_{\text{OXA-48}}$	OXA 48F- TTGGTGGCATCGATTATCGG	(Armand-Lefèvre et al., 2003)
	OXA 48R- GAGCACTTCTTTTGTGATGGC	
$\textit{bla}_{\text{TEM-1}}$	TEM A1- ATAAAATTCTTGAAGAC	(Speldooren et al., 1998)
	TEM B1- TTACCAATGCTTAATCA	
ompK35	ompk35 cplt F- CAGACACCAAACTCTCATCA	This study
	ompk35 cplt R- TCAGGTTAACGGGAGAATAA	
	ompk35seqF- GCGTCGATCAGAAAGCT	
	ompk35seqR- GCTTCGGCTTTGTCGCCA	
	ompk35 promF- GATTAAGTTCACAAAGTTCCG	
ompK36	ompk36 cplt F- TTGCAGCACAATGAAATAGC	This study
	ompk36 cplt R- CAAGAGTATACCAGCGAGGT	
	ompk36 Seq F- CGACGTTCTGCCGGAATT	
	ompk36 Sqce-R- AGAGTTACGGTAGGTTGC	
	ompK36 promF- ACATCTTGTGGGAACTTTGAA	
	ompK36-IS F- CACCAATGTGGGCCTTCATG	
	ompK36-IS R- TCATGAGCCATCAACTCACC	

Table S1. List of bacterial strains, plasmids and primers used in this study (continued)

Strain, plasmid or primer	Description	Reference or source
ramR	ramR F- CACGGTTCATATCCTGACCA	(Bialek-Davenet et al., 2011)
	ramR R- CCRTCGACCTTAAACACGTC	
Knockout		
acrB	acrBForFRTKm-<br>TCTGATGATAAACAGCAAGCCGCAGGCAACGCGCCATCAGAACAAACCAAGTCTT<br>AACTTAAACAGGAGCCGTTAAGACGTGTAGGCTGGAGCTGCTTC	(Bialek-Davenet et al., 2015)
	acrBRevFRTKm-<br>AACAATGGCAATATGTGCCTTGCCAGCCAGTGATAAAAAAGGGCCGCGGTAGCGG<br>CCCTTTGTTCAGGAGTGAAGACATATGAATATCCTCCTTAGTTCC	(Bialek-Davenet et al., 2015)
	acrBupF- GGATGTCACCGTCGATCAG	(Bialek-Davenet et al., 2015)
	acrBupR- CCGACAACCATCAGGAAGCT	
	acrBdoF- TCTGCTGACCACCATCGGCCT	(Datsenko and Wanner, 2000)
	acrBdoR- GGCAGGCAGCTGTCGCGTTC	
	k2- CGGTGCCCTGAATGAACTGC	
	kt- CGGCCACAGTCGATGAATCC	
ramA	ramAFRTKM-F<br>GGTGTGGGTCGCCGATAAGACGCAATCATTTAACGCCTGGTGGCGCTAAGCGCCAG<br>TGCAGTATCAGAGGAGAGAGCATGTGTAGGCTGGAGCTGCTTC	This study
	ramAFRTKM-R<br>CATTGAGTATCTGGTGCTGCGCTGGGTGTGGCGTCGCTGGTTTTCCCTCGAGCGGTA<br>AACCAGGAGAGTCGCGCCCATATGAATATCCTCCTTAGTTCC	(Bialek-Davenet et al., 2011)
	Loc ram F- TGGGATGAACCGTATCAACG	(Datsenko and Wanner, 2000)
	Loc ram R- ATCTTACTGCTGGCCCTGCT	
	K1- CAGTCATAGCCGAATAGCCT	
Cloning		
oqxB	XbaI-oqxRF- GCATCTCTAGATTCCTGACGCCGGTGTTTTA	This study
	XhoI-oqxRR- GATATCTCGAGCTGCGGTGCCAAAAAGAACA	
RT-PCR		
ompK35	ompk35RT F- TGATCCCTGCCCTGCTGGT	This study
	ompk35RT R- TCCATGTTGTATTCCCACTGG	
ompK36	ompk36RT F- GCGACCAGACCTACATGCGT	This study
	ompk36RT R- AGTCGAAAGAGCCCGCGTC	
rarA	rarA-RT-F- TGGATCGACAACCATCTTGA	(Veleba et al., 2013)
	rarA-RT-R- AAGGACTGCTGGGAGTCAAA	
oqxB	oqxB1806F- GAGCGAGATCGGGATGAATAC	(Bialek-Davenet et al., 2015)
	oqxB1890R- CGGCGTGTTGGTGAACTG	
ramA	ramA-F- ATCGTCGAGTGGATTGATGA	(Bratu et al., 2009)
	ramA-R- AGATGCCATTTCGAATACCC	
acrB	acrB-F- CGATAACCTGATGTACATGTCC	(Doumith et al., 2009)
	acrB-R- CCGACAACCATCAGGAAGCT	
rpoB	rpoBF- AAGGCGAATCCAGCTTGTTCAGC	(Bialek-Davenet et al., 2011)
	rpoBR- TGACGTTGCATGTTCGCACCCATCA	

Kan, kanamycin; Amp, ampicillin; Tet, tetracycline.

References

Armand-Lefèvre, L., Leflon-Guibout, V., Bredin, J., Barguellil, F., Amor, A., Pagès, J. M., et al. (2003). Imipenem resitance in *Salmonella enterica* serovar Wien related to porin loss and CMY-4 β-lactamase production. *Antimicrob. Agents Chemother.* 47, 1165–1168.

Balestrino, D., Haagensen, J. A. J., Rich, C., and Forestier, C. (2005). Characterization of type 2 quorum sensing in *Klebsiella pneumoniae* and relationship with biofilm formation. *J. Bacteriol.* 187, 2870–2880. doi:10.1128/JB.187.8.2870-2880.2005.

Bialek, S., Lavigne, J. P., Chevalier, J., Marcon, E., Leflon-Guibout, V., Davin, A., et al. (2010). Membrane efflux and influx modulate both multidrug resistance and virulence of *Klebsiella pneumoniae* in a *Caenorhabditis elegans model*. *Antimicrob. Agents Chemother.* 54, 4373–4378.

Bialek-Davenet, S., Lavigne, J.-P., Guyot, K., Mayer, N., Tournebize, R., Brisse, S., et al. (2015). Differential contribution of AcrAB and OqxAB efflux pumps to multidrug resistance and virulence in *Klebsiella pneumoniae*. *J. Antimicrob. Chemother.* 70, 81–88.

Bialek-Davenet, S., Leflon-Guibout, V., Tran Minh, O., Marcon, E., Moreau, R., and Nicolas-Chanoine, M.-H. (2013). Complete deletion of the *ramR* gene in an *in vitro*-selected mutant of *Klebsiella pneumoniae* overexpressing the AcrAB efflux pump. *Antimicrob. Agents Chemother.* 57, 672–673.

Bialek-Davenet, S., Marcon, E., Leflon-Guibout, V., Lavigne, J. P., Bert, F., Moreau, R., et al. (2011). *In vitro* selection of *ramR* and *soxR* mutants overexpressing efflux systems by fluoroquinolones as well as cefoxitin in *Klebsiella pneumoniae*. *Antimicrob. Agents Chemother.* 55, 2795–2802.

Bratu, S., Landman, D., George, A., Salvani, J., and Quale, J. (2009). Correlation of the expression of *acrB* and the regulatory genes *marA*, *soxS* and *ramA* with antimicrobial resistance in clinical isolates of *Klebsiella pneumoniae* endemic to New York City. *J. Antimicrob. Chemother.* 64, 278–283.

Datsenko, K. A., and Wanner, B. L. (2000). One-step inactivation of chromosomal genes in *Escherichia coli* K-12 using PCR products. *Proc. Natl. Acad. Sci. U. S. A.* 97, 6640–6645. doi:10.1073/pnas.120163297.

Doumith, M., Ellington, M. J., Livermore, D. M., and Woodford, N. (2009). Molecular mechanisms disrupting porin expression in ertapenem-resistant *Klebsiella* and *Enterobacter* spp. clinical isolates from the UK. *J. Antimicrob. Chemother.* 63, 659–667.

Kovach, M. E., Elzer, P. H., Hill, D. S., Robertson, G. T., Farris, M. A., Roop, R. M., et al. (1995). Four new derivatives of the broad-host-range cloning vector pBBR1MCS, carrying different antibiotic-resistance cassettes. *Gene* 166, 175–176.

Pai, H., Kang, C.-I., Byeon, J.-H., Lee, K.-D., Park, W. B., Kim, H.-B., et al. (2004). Epidemiology and clinical features of bloodstream infections caused by AmpC-type-

beta-lactamase-producing *Klebsiella pneumoniae*. *Antimicrob. Agents Chemother.* 48, 3720–3728. doi:10.1128/AAC.48.10.3720-3728.2004.

Skerman, V. B. D., McGowan, V., and Sneath, P. H. A. eds. (1989). *Approved Lists of Bacterial Names (Amended)*. Washington (DC): ASM Press Available at: http://www.ncbi.nlm.nih.gov/books/NBK814/ [Accessed October 11, 2017].

Speldooren, V., Heym, B., Labia, R., and Nicolas-Chanoine, M.-H. (1998). Discriminatory detection of inhibitor-resistant β-lactamases in *Escherichia coli* by single strand conformational polymorphism-PCR. *Antimicrob. Agents Chemother.* 42, 879–884.

Veleba, M., De Majumdar, S., Hornsey, M., Woodford, N., and Schneiders, T. (2013). Genetic characterization of tigecycline resistance in clinical isolates of *Enterobacter cloacae* and *Enterobacter aerogenes*. *J. Antimicrob. Chemother.* 68, 1011–1018.
